# Improved distribution of leg forces after fibular resection and synostosis

**DOI:** 10.1186/s40634-022-00480-0

**Published:** 2022-05-16

**Authors:** Tariq Rahman, Geovanny Oleas-Santillan, Jinyong Wee, William G. Mackenzie

**Affiliations:** 1 Department of Biomedical Research, Nemours Children’s Hospital Delaware, 5424 E400 Experimental Station, 200 Powder Mill Road, Wilmington, DE 19803 USA; 2 Department of Orthopaedics, Nemours Children’s Hospital Delaware, 1600 Rockland Road, Wilmington, DE 19803 USA

**Keywords:** Genu varum, Strain, Ankle valgus, Tibiofibular synostosis

## Abstract

**Purpose:**

Genu varum- a common symptom in achondroplasia- may lead to ankle valgus in children. Ankle valgus may be mitigated by tibiofibular synostosis, but this is not always the case. The mechanical environment around the growth plates plays an important role in ankle valgus. The purpose of this project is 1) to determine the strain through the distal fibular growth plate before and after tibiofibular synostosis, and 2) postulate whether the change in strain affects ankle valgus. This project measured the distal fibular strain in a porcine hind leg model.

**Methods:**

The lower legs of seven pigs were removed, instrumented with strain gauges, and loaded compressively in a material testing machine. Loads were applied at three phases: 1) the intact leg, 2) leg with fibula resected, and 3) leg with fibula resected and tibiofibular synostosis. Mean strains were compared between phases using a mixed affect model. The significance level was adjusted for multiple comparisons using the Bonferroni method.

**Results:**

Phase 1, intact leg, had the highest strain value at 1247.9μɛ. In phase 2, the mean strain was 106.2μɛ. In phase 3, the compressive mean strain dropped to 477.4μɛ, which is 38% of the strain in phase 1. Standard error was 86.8μɛ; *p* < 0.001.

**Conclusion:**

Results indicate that more of the force through the leg is transmitted through the tibia after fibular resection and tibiofibular synostosis, which improves the balance of forces through the leg.

**Supplementary Information:**

The online version contains supplementary material available at 10.1186/s40634-022-00480-0.

## Background

Genu varum is common in achondroplasia and may result in waddling gait, impaired joint function, gait limitation, and pain [17]. The etiology is not known but includes asymmetric endochondral ossification, laxity of the lateral collateral ligament, and overgrowth of the fibula [9, 17, 14].

A historic procedure to correct for fibular overgrowth in genu varum is resection of the proximal fibula. This procedure has been shown to be successful in 64% of the cases in children [18]. It is thought to reduce the ‘thrust’ imparted on the distal fibula from the proximal fibular physis [2, 5] and alleviate the issue of higher fibular-to-tibial length ratio, bringing it in line with normal values [11]. This, however, may result in ankle valgus. Langenskiöld [8] first proposed the distal tibiofibular synostosis procedure to stop the distal fibula migrating proximally and preventing ankle valgus caused by fibular pseudoarthrosis. However, with the distal fibula rigidly connected, it can no longer move axially and laterally to provide stability to the ankle mortice and act as a shock absorber in concert with the interosseous membrane [19]. Kanaya and colleagues [6] described this procedure for children who developed ankle valgus after fibular grafts and noted that tibiofibular synostosis can impede the development of ankle valgus; however, it cannot completely prevent its development [4, 10]. Tibiofibular synostosis may prevent severe ankle valgus; however, the loss of the normal mechanics of the distal tibiofibular-talar articulation alters the growth pattern, leading to gradual valgus deformity in a growing child. There is a paucity of information on the quantity of load being transmitted through the tibia and remnant fibula after tibiofibular synostosis [3]. Information on the ratio of tibial to fibular forces before and after tibiofibular synostosis may help in understanding the etiology of ankle valgus. The aim of this study is to determine the strain through the fibula before and after the tibiofibular synostosis.

## Methods

### Porcine model

The strains in the distal fibula proximal to the epiphysis in seven cadaveric porcine hind legs under compressive loading were measured. This was performed in three phases: a) in the intact lower leg, b) after fibular resection, c) after tibiofibular synostosis (Fig. 2). The strains going through the fibula will be altered following the procedure.

The porcine specimens were obtained from adolescent Yorkshire pigs weighing 32-34 kg. The specimens were stored in the freezer and thawed at room temperature prior to the experiment. All procedures were preapproved by the institutional biosafety committee and performed in a Biosafety Level 1 biomechanics lab.

### Experimental procedure

Each porcine leg was first removed from the body of the pig. Then each leg below the knee was salvaged for the experiment. All the soft tissue on the leg was left intact including the interosseus membrane. The foot was secured on to a ¾” wooden board with screws applied from the underside of the board into the calcaneus and forefoot. To secure the proximal tibia, screws were placed into the tibial plateau, which allowed embedding the exposed screws into a fiberglass resin (Bondo, 3 M Corp). The leg was then turned upside down, and the exposed screws were positioned in a polyvinyl chloride (PVC) cup. The top (wooden board) and bottom (PVC cup) were then leveled with a spirit level while ensuring that the leg was close to vertical. The liquid resin was then poured into the cup and allowed to cure.

**Fig. 1:**
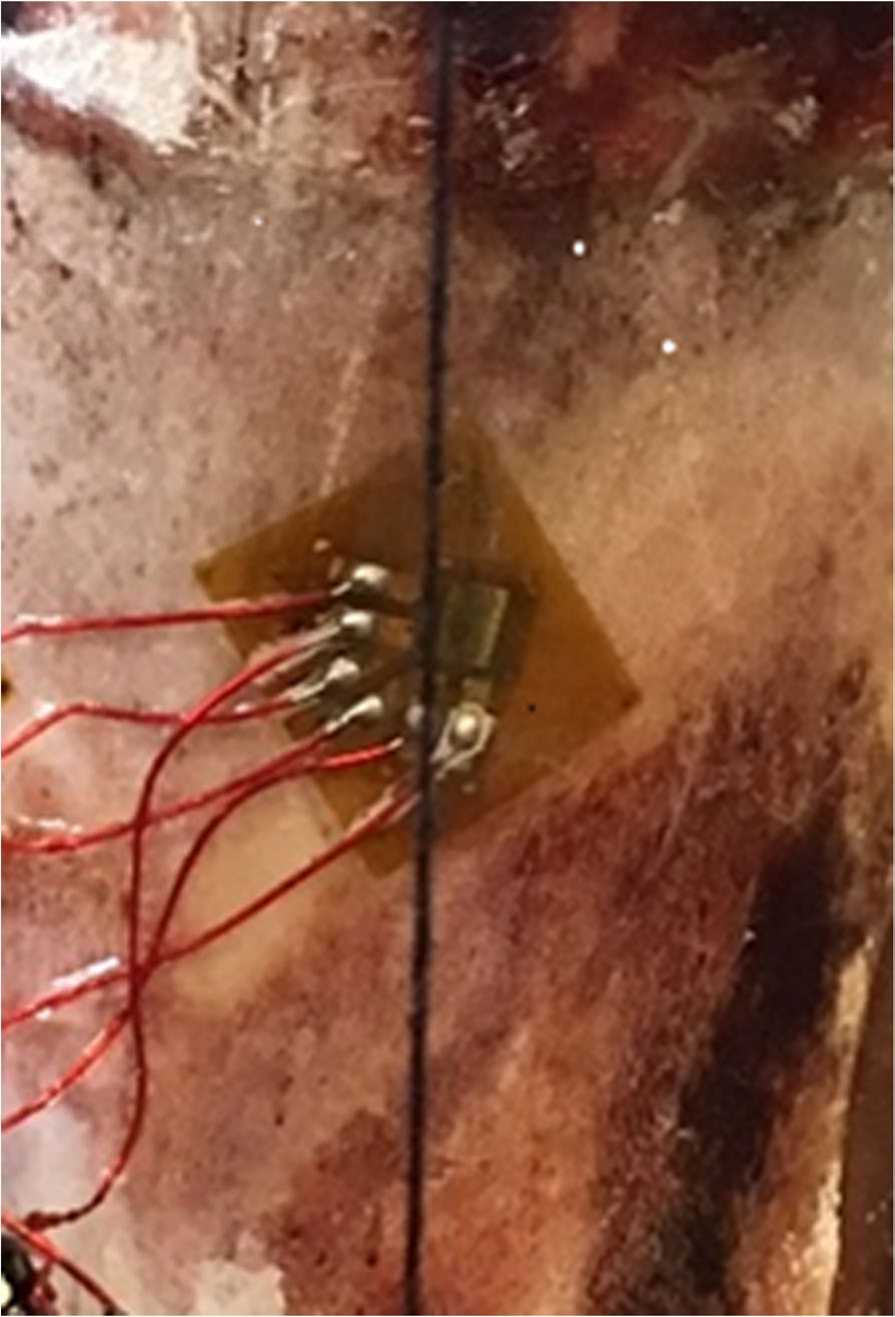
The stacked rosette strain gauge shown attached to the distal fibula

An incision was made approximately 2 cm proximal to the distal fibular physis, and an area for the strain gauge application was prepared. After the periosteum was removed, the area was sanded and cleaned with solvent. A pre-wired stacked rosette strain gauge (C2A-13-031WW-120, Micro-Measurements Group, Raleigh, NC) was bonded (M-Bond 200, Micro-Measurements Group) to the bone and lined up along the length of the fibula (Fig. 1). The gauge was then sealed with a coat of polyurethane. The gauge was tested to ensure the appropriate readings were obtained. The strain gauge was connected to an amplifier/signal conditioner (iNET-420, Omega Engineering Inc.) that was connected to a personal computer (PC) using instruNet software (Omega Engineering Inc.). A plum line was taped to the top of the PVC cup with the plumb coinciding with a fixed point on the wooden board to ensure that the specimen was always in the same vertical orientation when placed in the material testing machine.

**Fig. 2:**
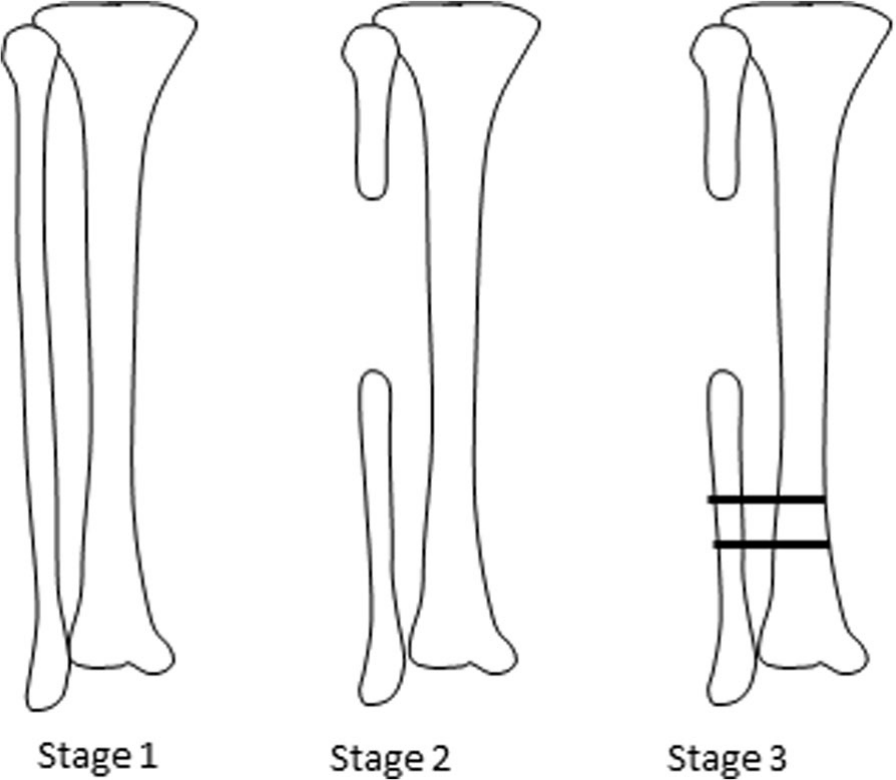
The three phases of the procedure with fibular resection and synostosis

**Fig. 3:**
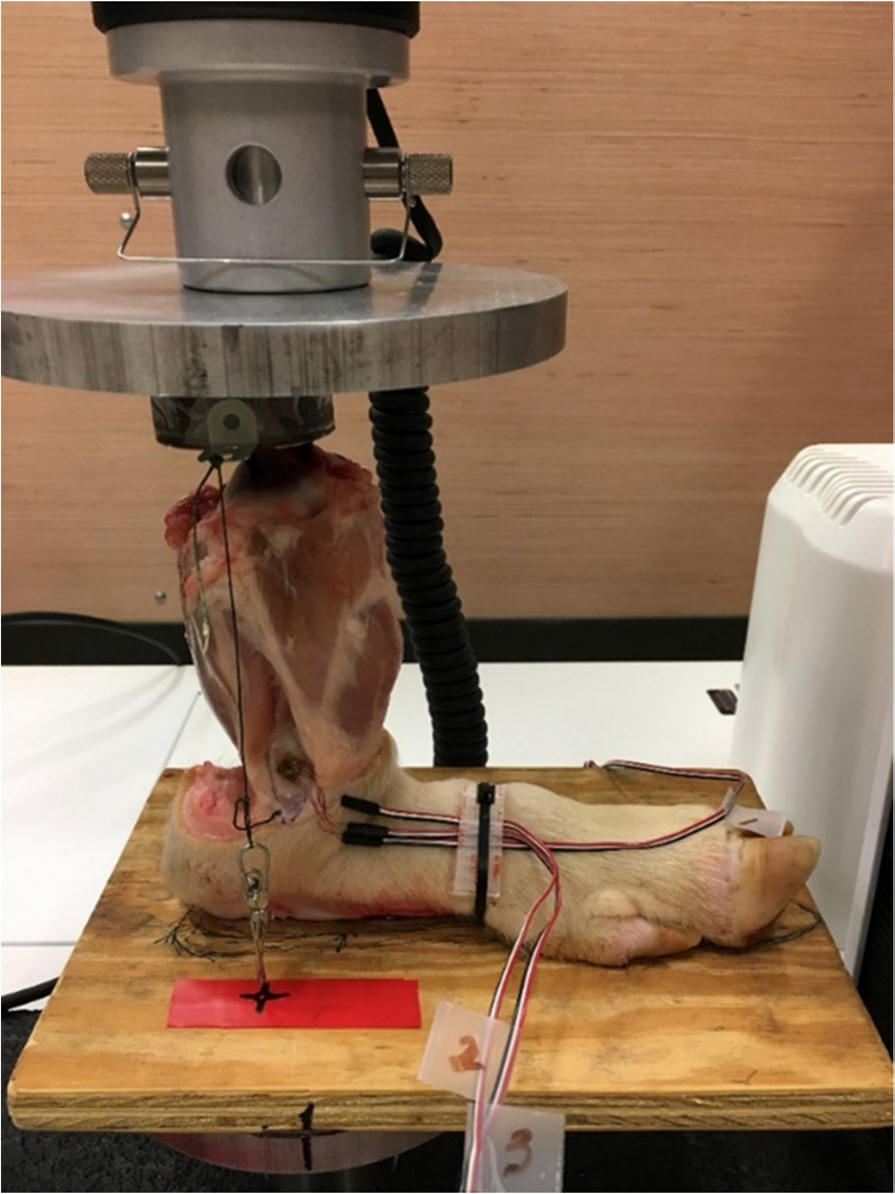
The pig leg undergoing compressive loading with plumb line suspended at the top

The testing was performed in three consecutive phases (Fig. 2). In phase 1, the leg was intact. In phase 2, a 1-cm section of the fibula was removed at the metaphysis with a bone saw. In phase 3, the distal remnant of the fibula was secured with two screws to the tibia to create a tibiofibular synostosis. After phase 1 testing, two holes were drilled in the distal fibula and tibia approximately 1 cm above the strain gauge to place the screws for phase 3. Then, the fibula was resected. The specimen was placed in the material testing machine (Model 3366, Instron, Norwalk CT) (Fig. 3), and the position of the board was marked on the base plate of the Instron machine, so the position of the specimen was always consistent. The Instron machine was controlled through a PC using Bluehill Universal software (Instron).

The Instron machine applied compressive loading by operating in displacement mode at a rate of 0.5 mm/min until the compressive force reached 25 kg. The force limit was well within normal ground reaction forces on each leg during dynamic movement, which could reach a peak of three times body weight while running [13]. Instron data were collected at 100 Hz, and the strains from the gauges were collected at 10 Hz. The strain gauge readings were zeroed before each trial. Each pig leg was tested 4 times for each of the 3 phases to equal 12 data sets per leg. The average of the 4 times was used as the final result. The maximum strain gauge reading was obtained for each trial. These readings correspond to the 25 kg compressive load.

### Statistical analysis

The outcome measure was maximum strain through the fibula. To compare the mean strain between phases, a mixed effects model with least square estimated mean was used. Phase was used as the fixed effect, and pig number was used as the random effect in the model to account for heterogeneity. The Bonferroni method was used to adjust the significance level for multiple pairwise comparisons. Statistical significance was set at *p* < 0.05. The statistical software SAS version 9.4 was used for the analysis.

## Results

**Fig. 4:**
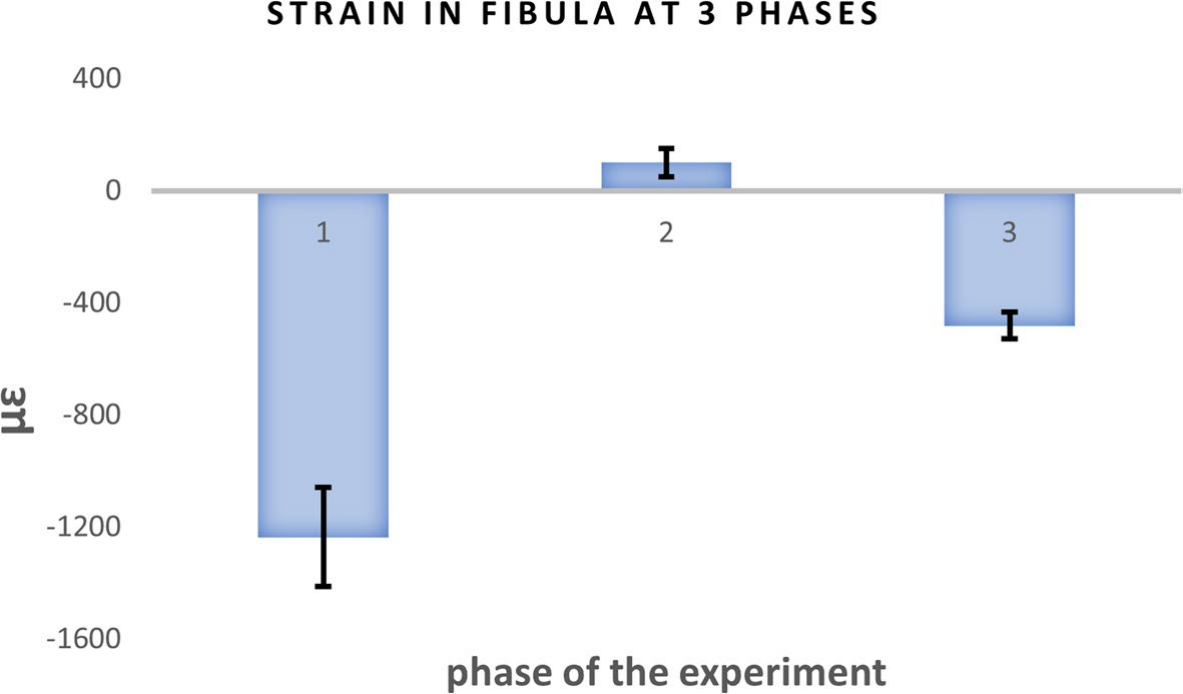
Graph of fibular strain in the three phases of the procedure. Standard error is 86.8μɛ; *p* < 0.001

Data show that phase 1, intact leg, has the highest compressive strain value at 1247.9μɛ (all *p*-values < 0.001). In phase 2, the mean strain is 106.2μɛ and is in tension, which is indicative of the force transmission through the interosseous membrane. In phase 3, the compressive mean strain drops to 477.4μɛ, which is 38% of the strain in phase 1 (Fig. 4). This indicates that more of the force through the leg is transmitted through the tibia after fibular resection and tibiofibular synostosis. Pairwise comparisons are shown in Table 1. The means for all the strain values for each phase were calculated.

**Table 1:** Strain gauge results shown in μɛ (SE = standard error)

Pairwise difference	Mean diff (SE)	*p*-value
phase 1-phase 2	− 1354.1 (54.5)	< 0.001
phase 1-phase 3	− 770.4 (54.5)	< 0.001
phase 2-phase 3	583.6 (54.5)	< 0.001

## Discussion

The current study has shown that at 25 kg loading of the leg, the strain in the intact fibula is 1247 μɛ (in compression). With the fibula resected, the strain drops to 106μɛ (in tension), and after connecting the distal fibular remnant to the tibia, the strain goes to 477μɛ (in compression). After resection and synostosis, the strain drops to 38% of the strain with an intact fibula. Clinically, the significance of this result is that there will be a better distribution of forces at the ankle with synostosis than without which will lead to less valgus at the ankle.

The load carried by the intact fibula is reported to be approximately 16.7% of the total load [7] carried by the leg. This means that the load carried by the fibula after phase 3 is 6.4% of the total load. A similar study [3] placed pylon force transducers in the fibula and tibia and applied compressive loading to human cadaveric specimens. Although this study did not resect the bones to measure load, the results are similar to the present study. Goh et al. [3] reported that the load on the fibula was 2.8% of the total force when the ankle was in neutral position. However, the force they measured transmitted through the intact fibula was only 7.1%.

The success in bringing the leg into alignment and preventing ankle angulation through fibular resection and tibiofibular synostosis is mixed [2, 4, 6]. Quantifying the load sharing between the two bones after both procedures may explain the reasons for this success. Proximal fibular resection has been used to treat genu varum in growing children and is successful in about 64% of the cases [18]. The mechanism of correction is not clear. After the Langenskiöld procedure [8], ankle valgus can progress, albeit at a diminished rate. In a retrospective study [4], tibiotalar tilt angle (range 5°–30°) was found in 11 of 23 cases (45%) following fibulectomy in patients in their growth period. The tibiofibular joint was stabilized with a suprasyndesmal screw or a Kirchner wire in half of the cases.

Two studies [9, 17] have shown that fibular to tibial length ratio is significantly related to the alignment indices of the lower limb. and that the fibular growth rate exceeds the tibial growth rate in achondroplastic patients during their formative years. However, other studies do not demonstrate that a long fibula is always present in genu varum in these children [1].

The Langenskiöld procedure has been the accepted treatment modality for ankle valgus [6, 8, 12]. However, more recent articles have shown ankle valgus occurring with or without synostosis [2, 4, 6]. This may be due to the biomechanical changes between the tibia and the fibula after a synostosis and can lead to valgus angulation. The present study attempts to answer some of these biomechanical questions by quantifying the forces after the procedure. It has been shown that synostosis leads to an improved distribution of forces at the ankle.

Investigations using roentgenography [15] and cineroentgenography [19] have shown that the fibula moves distally during weight bearing to deepen the ankle mortise, thus providing greater stability. Fibular movement is permitted by the orientation of the fiber of the interosseous membrane [3]. Skraba et al. [16] found that the interosseous membrane plays a critical role in the load-sharing ability of the fibula. After incision of the membrane, fibular strains decreased to essentially zero, thus supporting the hypothesis that the interosseous membrane acts as a conduit for stress transmission to the fibula. This study supported results in the present study, as the load transmitted through the distal segment of the fibula after resection (phase 2) did not yield zero strain but, in fact, was 1.4% of the total leg force. The fibula acts as a shock absorber and enables some of these forces to be dissipated by translating them into tension forces in the interosseous membrane and tibiofibular ligaments.

Frick et al. [2] noted progressive shortening of the lateral malleolus with mild to moderate ankle valgus with tibiofibular synostosis. Synostosis may prevent severe deterioration of ankle valgus; however, the loss of the normal mechanics of the distal tibiofibular-talar articulation alters the growth pattern, leading to gradual valgus deformity in a growing child. Given the abnormal ankle mechanics and the potential for ankle pain with loss of normal tibiofibular motion, Martus et al. [12] recommend synostosis only for failed fibular osteosynthesis or severe valgus with a short distal fibular segment.

One of the current study’s shortcomings is the use of cadaveric porcine hind legs versus human cadaver legs. The porcine legs were readily available as they were harvested from an unrelated concurrent study. The pig hind legs include a tibia and fibula and offered a good model to measure the difference between pre- and post-procedure. Also, this study did not include multiple strain gauges around the distal fibula and tibia. Additional gauges would provide a complete picture of the strains around the bones including bending moments. This would allow the uncoupling of the bending from the axial loads, particularly in the fibula.

## Conclusions

The strain through a series of porcine fibulae after resection and tibiofibular synostosis was shown to be less than 50% of that of the intact leg. Therefore, more of the force through the leg is going through the tibia after the procedure. This results in a more stable distribution of forces at the ankle.

## Supplementary Information

**Additional file 1.**

## Data Availability

Provided in supplemental file.

## Abbreviations

PVC: Polyvinyl chloride
PC: Personal computer
